# Structure Identification and Anti-Cancer Pharmacological Prediction of Triterpenes from *Ganoderma lucidum*

**DOI:** 10.3390/molecules21050678

**Published:** 2016-05-21

**Authors:** Yanyan Shao, Liansheng Qiao, Lingfang Wu, Xuefei Sun, Dan Zhu, Guanghui Yang, Xiaoxue Zhang, Xin Mao, Wenjing Chen, Wenyi Liang, Yanling Zhang, Lanzhen Zhang

**Affiliations:** 1 School of Chinese Materia Medica, Beijing University of Chinese Medicine, Beijing 100102, China; sunshine4003@126.com (Y.S.); fanglingwu@163.com (L.W.); sunxuefei.2008@163.com (X.S.); hbbdzhudan@163.com (D.Z.); yghui1990@163.com (G.Y.); zhangxiaoxue122@163.com (X.Z.); mxmaoxin@126.com (X.M.); sdcwjing@163.com (W.C.); lwy1054289310@163.com (W.L.); 2 Beijing Key Laboratory of TCM Foundation and New Drug Research, School of Chinese Material Medica, Beijing University of Chinese Medicine, Beijing 100102, China; b20100222012@163.com

**Keywords:** *Ganoderma lucidum*, lanostanoid triterpene, pharmacophore, protein interaction network, anti-cancer, reverse target identification

## Abstract

*Ganoderma* triterpenes (GTs) are the major secondary metabolites of *Ganoderma lucidum*, which is a popularly used traditional Chinese medicine for complementary cancer therapy. In the present study, systematic isolation, and *in silico* pharmacological prediction are implemented to discover potential anti-cancer active GTs from *G. lucidum*. Nineteen GTs, three steroids, one cerebroside, and one thymidine were isolated from *G. lucidum*. Six GTs were first isolated from the fruiting bodies of *G. lucidum*, including 3β,7β,15β-trihydroxy-11,23-dioxo-lanost-8,16-dien-26-oic acid methyl ester (**1**), 3β,7β,15β-trihydroxy-11,23-dioxo-lanost-8,16-dien-26-oic acid (**2**), 3β,7β,15α,28-tetrahydroxy-11,23-dioxo-lanost-8,16-dien-26-oic acid (**3**), ganotropic acid (**4**), 26-nor-11,23-dioxo-5α-lanost-8-en-3β,7β,15α,25-tetrol (**5**) and (3β,7α)-dihydroxy-lanosta-8,24-dien- 11-one (**6**). (4*E*,8*E*)-*N*-d-2′-hydroxypalmitoyl-l-*O*-β-d-glucopyranosyl-9-methyl-4,8-spingodienine (**7**), and stigmasta-7,22-dien-3β,5α,6α-triol (**8**) were first reported from the genus *Ganodema*. By using reverse pharmacophoric profiling of the six GTs, thirty potential anti-cancer therapeutic targets were identified and utilized to construct their ingredient-target interaction network. Then nineteen high frequency targets of GTs were selected from thirty potential targets to construct a protein interaction network (PIN). In order to cluster the pharmacological activity of GTs, twelve function modules were identified by molecular complex detection (MCODE) and gene ontology (GO) enrichment analysis. The results indicated that anti-cancer effect of GTs might be related to histone acetylation and interphase of mitotic cell cycle by regulating general control non-derepressible 5 (GCN5) and cyclin-dependent kinase-2 (CDK2), respectively. This research mode of extraction, isolation, pharmacological prediction, and PIN analysis might be beneficial to rapidly predict and discover pharmacological activities of novel compounds.

## 1. Introduction

*Ganoderma lucidum* (Leyss. ex Fr.) Karstis is one of the most highly used medicinal fungi in the world. Its fruiting body, called Lingzhi or Reishi, has been widely used in traditional Chinese medicine (TCM) as a dietary supplement and medicinal herb in China and other eastern countries for health promotion. Modern medical research has indicated that *G. lucidum* had comprehensive biological activities, such as anti-cancer, anti-inflammatory, immune-modulating, anti-oxidant, anti-microbial, anti-HIV-1, and so on, between which the most attractive is anti-cancer activity [1,2,3,4,5,6,7,8,9].

To date, over 400 compounds were isolated and identified from *G. lucidum*. Wherein, more than 150 compounds belonged to GTs which were regarded as the main medicinal components, such as ganoderic acid A (GA-A), GA-C_2_, GA-D, GA-DM, lactone, ganoderiol F, ganodermanotriol, and so on [10,11,12,13,14,15]. Accumulating evidence has shown that GTs can inhibit the proliferation of hepatoma cells and HeLa cells, as well as human colon cancer cells HT-29 [16,17,18]. However, the structures and pharmacological activities of some GTs from *G. lucidum* are still unknown.

Virtual screening is an effective method to predict the biological activity of compounds [19]. Pharmacophore-based activity profiling is a high-performance virtual screening method [20], which can discover potential therapeutic targets of compounds. Generally, TCM compounds with diversified structures have multi-targets effect [21,22]. Therefore, holistic analysis of the relationship and biological process of TCM therapeutic targets is beneficial to reveal the pharmacological activities of TCM compounds. Ingredient-target interaction network and protein interaction network (PIN) are two main types of bioinformatics methods to research the multi-target pharmacological effect of compounds. Ingredient-target interaction network is commonly constructed by experimental or predicted information of ingredient-target interactome, which can reflect the therapeutic targets of compounds directly. PIN is constructed by protein-protein interactions (PPIs), which refer to the major link of the biological process of therapeutic targets [23]. Meanwhile, module-based network analysis of PIN is able to explore the biological effect of TCM therapeutic targets and initially identify the pharmacological activities of a single or class of TCM compound [24,25].

Several *in silico* anti-cancer pharmacological prediction and profiling of GTs had been attempted. According to the docking studies of anti-cancer targets IKK1 and IKK2, Balraj [26] discovered GA-A and GA-H might have the potential anti-cancer activities for NF-κB signaling pathway. Anti-cancer mechanism of GA-D was also discussed by QingXi [27]. GA-D was docked into anti-cancer target-related proteins identified by cell experiments, and hit proteins were considered to interact with GA-D. PINs were then constructed to discuss the anti-cancer mechanism of GA-D and the contribution of these anti-cancer target-related proteins. Generally, existing research mainly focused on pharmacological prediction of single GT compound. However, the activity prediction of a class of GTs with similar structural framework and functional group should also be concerned.

In order to search for more active GTs from *G. lucidum*, studies on chemical constituents of *G. lucidum* and their biological activity prediction were carried out deeply and systematically. Solvent extraction and silica gel column chromatography were utilized to isolate the novel active constituents from the fruiting bodies of *G. lucidum*, including GTs, sterides and cerebrosides. Then, reverse target identification of novel isolated GTs was implemented by pharmacophore database and hit targets were used to construct ingredient-target network. Thereafter, high frequency targets from ingredient-target network were selected and their target-related proteins were utilized to construct PIN. According to the results of module analysis of PIN, anti-cancer modules were recognized and pharmacological activity of GTs was explained by gene ontology (GO) enrichment analysis.

## 2. Results and Discussion

### 2.1. Identification of the Compounds

The structures of the isolated compounds were elucidated from the data obtained from ^1^H- and ^13^C-NMR spectra. 3β,7β,15β-trihydroxy-11,23-dioxo-lanost-8,16-dien-26-oic acid methyl ester (**1**) [28], 3β,7β,15β-trihydroxy-11,23-dioxo-lanost-8,16-dien-26-oic acid (**2**) [28], 3β,7β,15α,28-tetrahydroxy-11,23-dioxo-lanost-8,16-dien-26-oic acid (**3**) [29], ganotropic acid (**4**) [29], 26-nor-11,23-dioxo-5α-lanost-8-en-3β,7β,15α,25-tetrol (**5**) [30], (3β,7α)-3,7-dihydroxylanosta-8,24- dien-11-one (**6**) [31] were first reported from the species of *G. lucidum*. Meanwhile, (4*E*,8*E*)-*N*-d-2′-hydroxypalmitoyl-l-*O*-β-d-glucopyranosyl-9-methyl-4,8-spingodienine (**7**) [32] and stigmasta-7,22-dien-3β,5α,6α-triol (**8**) [33] were first reported from the genus *Ganodema*, together with 4,4,14α-trimethyl-3-oxo-5α-pregna-7,9(11)-dien-20*S*-carboxylate (**9**), lucidenic acid C (**10**), lucidone F (**11**), lucidone D (**12**), 7-oxo-ganoderic acid Z (**13**), ganosineniol A (**14**), ganoderic acid XL_1_ (**15**), ganoderic acid C_2_ (**16**), methyl ganoderate C (**17**), ganolucidic acid γ_a_ (**18**), methyl ganoderate C_2_ (**19**), lucidenic lactone (**20**), ganodericacid C (**21**), ganodermacetal (**22**) and ergosterol (**23**), and ergosta-7,22-dien-3-one (**24**).

Compound **1** was obtained as a yellow oily solid, yielded a positive reaction to 10% H_2_SO_4_–EtOH reagent. ^1^H-NMR (CD_3_OD, 500 MHz) δ: 3.57 (3H, s, O-Me), 2.91 (1H, dd, *J* = 8.4, 17.8 Hz, H-3), 4.96 (1H, dd, *J* = 7.2, 9.9 Hz, H-7), 5.49 (1H, s, H-15), 5.44 (1H, s, H-16), 2.78 (1H, m, H-1), 1.61 (2H, m, H-2), 0.95 (1H, m, H-5), 1.96 (1H, dd, *J* = 7.6, 17.6 Hz, H-6), 3.12 (1H, d, *J* = 15.0 Hz, H-12), 3.02 (1H, d, *J* = 15.0 Hz, H-12), 2.50 (1H, m, H-20), 2.54 (1H, dd, *J* = 6.8,16.6 Hz, H-22), 2.40 (1H, m, H-24), 2.73 (1H, m, H-25), 1.25 (3H, s, H-18), 1.22 (3H, s, H-19), 1.02 (3H, d, *J* = 6.4 Hz, H-21), 1.11 (3H, d, *J* = 7.1 Hz, H-27), 1.03 (3H, s, H-28), 1.01 (3H, s, H-29), 1.02 (3H, s, H-30); ^13^C-NMR (CD_3_OD, 125 MHz) δ: 34.77 (C-1), 28.65 (C-2), 77.03 (C-3), 39.24 (C-4), 49.52 (C-5), 28.59 (C-6), 69.32 (C-7), 161.39 (C-8), 141.47 (C-9), 39.20 (C-10), 199.79 (C-11), 46.65 (C-12), 51.56 (C-13), 56.48 (C-14), 77.43 (C-15), 123.89 (C-16), 154.22 (C-17), 22.46 (C-18), 19.62 (C-19), 27.78 (C-20), 20.22 (C-21), 48.54 (C-22), 207.57 (C-23), 48.29 (C-24), 35.27 (C-25), 176.17 (C-26), 16.99 (C-27), 28.83 (C-28), 16.65 (C-29), 23.46 (C-30), 52.10 (C-O-Me).

Compound **2** was obtained as a yellow oily solid, yielded a positive reaction to 10% H_2_SO_4_–EtOH reagent. The structure was the same as that of compound **1**, except for the substituent of C-26, with a carboxy group on it. ^1^H-NMR (C_5_D_5_N, 500 MHz) δ: 3.11 (1H, m, H-3), 4.47 (1H, dd, *J* = 5.2, 8.9 Hz, H-7), 5.38 (1H, s, H-15), 5.21 (1H, s, H-16); ^13^C-NMR (CD_3_OD, 125 MHz) δ: 78.82 (C-3), 70.10 (C-7), 161.91 (C-8), 142.65 (C-9), 201.68 (C-11), 77.96 (C-15), 125.61 (C-16), 155.77 (C-17), 209.82 (C-23), 179.6 (C-26).

Compound **3** was obtained as a yellow oily solid, yielded a positive reaction to 10% H_2_SO_4_–EtOH reagent. The structure was the same as that of compound **1**, except for its more than a hydroxyl of C-28 and opposite stereoconfiguration of chiral C-15. ^1^H-NMR (C_5_D_5_N, 500 MHz) δ: 4.29 (1H, m, H-3), 5.09 (1H, m, H-7), 5.96 (1H, brs, H-15), 5.59 (1H, brs, H-16), 4.22 (1H, d, *J* = 10.5 Hz, H-28), 3.76 (1H, d, *J* = 10.5 Hz, H-28); ^13^C-NMR (C_5_D_5_N, 125 MHz) δ: 71.9 (C-3), 69.3 (C-7), 161.7 (C-8), 141.6 (C-9), 200.0 (C-11), 77.3 (C-15), 125.8 (C-16), 154.6 (C-17), 210.3 (C-23), 183.2 (C-26), 66.6 (C-28).

Compound **4** was obtained as a white amorphous power, yielded a positive reaction to 10% H_2_SO_4_–EtOH reagent. The structure was possessed two oxygenic five-membered rings system in the side chain of lanostane skeleton. ^1^H-NMR (C_5_D_5_N, 500 MHz) δ: 3.20 (dd, *J* = 6.0, 11.6 Hz, H-3α), 4.37 (1H, d, *J* = 6.6 Hz, H-7α), 5.30 (1H, m, H-15β), 2.28 (1H, m, H-20), 1.85 (1H, d, *J* = 13.5 Hz, H-22α), 2.83 (dd, *J* = 8.0, 14.7 Hz, H-22β), 2.62 (dd, *J* = 6.4, 13.8 Hz, H-24α), 2.08 (1H, m, H-24β), 2.87 (1H, m, H-25), 1.21 (3H, d, *J* = 7.1 Hz, H-27); ^13^C-NMR (C_5_D_5_N, 125 MHz) δ: 77.43 (C-3), 69.09 (C-7), 161.12 (C-8), 141.77 (C-9), 200.24 (C-11), 72.83 (C-15), 45.54 (C-16), 95.21 (C-17), 44.05 (C-20), 17.97 (C-21), 44.50 (C-22), 113.29 (C-23), 44.57 (C-24), 35.66 (C-25), 178.64 (C-26), 15.01 (C-27).

Compound **5** was obtained as a white solid, yielded a positive reaction to 10% H_2_SO_4_–EtOH reagent. The structure was the same as that of compound **1**, except for its the degradation of the carboxyl group and the attachment of a hydroxyl group to C-25 and opposite stereoconfiguration of chiral C-15, with its more than two H atoms of C-16 and C-17. ^1^H-NMR (DMSO-*d*_6_, 500 MHz) δ: 3.20 (1H, dd, *J* = 5.2, 11.0 Hz, H-3), 4.42 (1H, dd, *J* = 7.3, 9.8 Hz, H-7), 4.68 (1H, t, *J* = 15.8 Hz, H-15), 4.12 (1H, m, H-25), 1.81 (2H, m, H-16); ^13^C-NMR (DMSO-*d*_6_, 125 MHz) δ: 78.07 (C-3), 67.39 (C-7), 160.72 (C-8), 140.89 (C-9), 199.45 (C-11), 70.5 (C-15), 36.57 (C-16), 47.89 (C-17), 213.84 (C-23), 76.39 (C-25).

Compound **6** was obtained as a white amorphous powder, yielded a positive reaction to 10% H_2_SO_4_–EtOH reagent. The structure was most likely a lanostane triterpenoid with the presence of a trisubstituted C=C bond at C-24. ^1^H-NMR (C_5_D_5_N, 500 MHz) δ: 3.46 (1H, dd, *J* = 7.8,12.7 Hz, H-3), 3.48 (1H, d, *J* = 9.5 Hz, H-7), 5.23 (1H, t, *J* = 6.6 Hz, H-24), 2.97 (1H, dd, *J* = 8.6, 14.9 Hz, H-1), 1.67 (2H, m, H-2), 1.73 (2H, m, H-6), 1.87 (1H, m, H-15), 1.73 (1H, m, H-15), 2.65 (1H, d, *J* = 15.7 Hz, H-12), 2.56 (1H, d, *J* = 15.7 Hz, H-12), 2.00 (2H, m, H-16), 1.88 (1H, m, H-23), 1.62 (3H, s, H-26), 1.51 (3H, s, H-27); ^13^C-NMR (C_5_D_5_N, 125 MHz) δ: 77.48 (C-3), 69.35 (C-7), 160.42 (C-8), 141.78 (C-9), 199.93 (C-11), 29.98 (C-15), 27.84 (C-16), 50.10 (C-17), 23.50 (C-23), 123.88 (C-24), 135.10 (C-25), 23.50 (C-26), 17.42 (C-27). 

Compound **7** was obtained as a white amorphous powder, yielded a positive reaction to 10% H_2_SO_4_–EtOH reagent. ^1^H-NMR (C_5_D_5_N, 500 MHz) δ: 8.32 (1H, d, *J* = 7.8 Hz, 2-NH), 6.02 (1H, m, 3-OH), 3.83 (1H, m, H-1), 4.86 (1H, d, *J* = 7.8 Hz, H-3), 4.50 (1H, t, H-4), 5.28 (1H, m, H-5), 3.97 (1H, m, H-8), 1.59 (3H, s, 9-CH3), 6.00 (1H, m, 2′-OH), 0.84 (6H, m, H-18, 16′, terminal methyls); ^13^C-NMR (C_5_D_5_N, 125 MHz) δ: 14.23 (C-1), 30.27, 29.99, 29.99, 29.97, 29.97, 29.89, 29.89 (C-2-8), 32.08 (C-9), 124.06 (C-10), 131.8 (C-11), 35.56 (C-12), 33.00 (C-13), 132.33 (C-14), 135.58 (C-15), 72.2 (C-16), 54.45 (C-17), 70.04 (C-18), 32.08 (C-19), 14.23 (C-1′), 16.01 (C-2′), 29.86, 29.86, 29.83, 29.78, 29.58, 29.30, 28.26, 28.11, 25.82, 22.89, 22.77 (C-3′-13′), 39.93 (C-14′), 72.39 (C-15′), 175.66 (C-16′), 105.54 (C-1′′), 75.00 (C-2′′), 78.31 (C-3′′), 71.36 (C-4′′), 78.44 (C-5′′), 62.50 (C-6′′). 

Compound **8** was obtained as a white solid, which when sprayed with 10% H_2_SO_4_ ethanol solution followed by heating showed a blue purple color. ^1^H-NMR (DMSO-*d*_6_, 500 MHz) δ: 5.07 (1H, m, H-7), 5.23 (1H, m, H-22), 5.16 (1H, dd, *J* = 15.3, 8.1 Hz, H-23), 4.21 (1H, d, *J* = 5.6 Hz, OH-3), 3.75 (1H, m, H-3), 3.58 (1H, s, OH-5), 4.48 (1H, d, *J* = 5.5 Hz, OH-6), 3.36 (1H, brs, H-6), 2.02–1.82 (6H, m, H-2, 9, 12, 20, 24), 1.82–1.76 (1H, m, H-14), 1.68–1.62 (2H, m, H-16 ), 1.60–1.20 (11H, m, H-1, 2, 4, 11, 15, 17, 25), 0.53 (3H, s, H-18), 0.88 (3H, s, H-19), 0.98 (3H, d, *J* = 6.6 Hz, H-21), 0.79 (3H, d, *J* = 7.2 Hz, H-26), 0.87 (3H, d, *J* = 6.6 Hz, H-27 ), 0.80 (3H, d, *J* = 7.2 Hz, H-28); ^13^C-NMR (DMSO-*d*_6_, 125 MHz) δ: 21.29 (C-1), 40.17 (C-2), 65.92 (C-3), 31.15 (C-4), 74.43 (C-5), 72.09 (C-6), 119.41 (C-7), 139.62 (C-8), 42.23 (C-9), 36.61 (C-10), 32.42 (C-11, C-25), 38.93 (C-12), 42.95 (C-13), 54.13 (C-14), 22.55 (C-15), 27.67 (C-16), 55.28 (C-17), 12.02 (C-18), 17.65 (C-19), 40.00 (C-20), 20.94 (C-21), 135.34 (C-22), 131.34 (C-23), 41.96 (C-24), 19.70 (C-26), 19.42 (C-27), 17.24 (C-28).

### 2.2. Pharmacophoric Profiling of GTs

**Figure 1:**
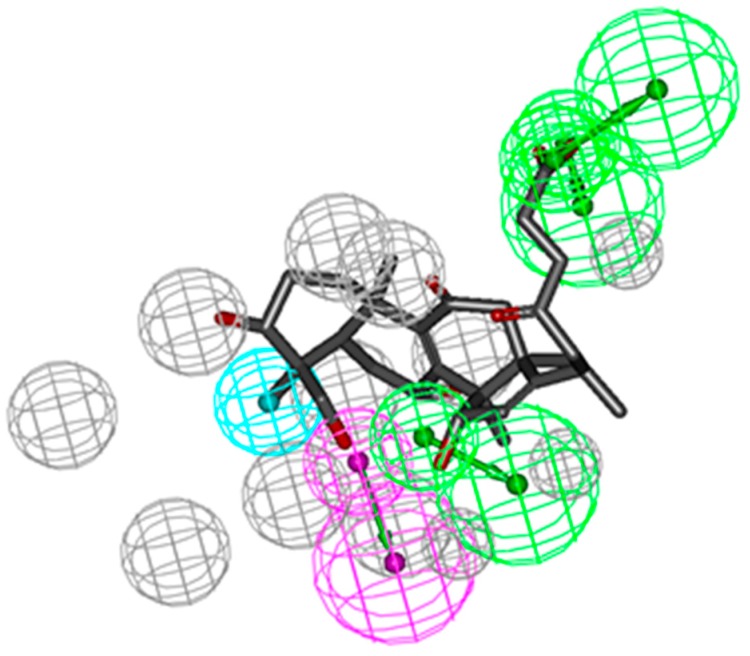
CDK2 SBP model (PDB: 1KE5) and molecular mapping of the Compound **3**.

Reverse pharmacophoric profiling of six isolated GTs and two positive GTs (GT-A and GT-D) was implemented to explore the pharmacological activity of GTs. In total, the reverse profiling results of eight GTs contained 30 human targets, and targets from the other creatures were eliminated because of less correlation with cancer. Top 20 interaction information of GTs and targets were showed in Table 1, based on the hit number of SBP models and fit values. Compound **1**, **2**, **3**, and **5** and two positive GTs could match with CDK2 pharmacophore model (PDB: 1KE5) with fit value more than 0.5, which indicated they might have better biological action to CDK2. The superior mapping result of compound **3** and the best model was showed in Figure 1. In general, the structural framework of the eight GTs is oxygenated lanostane, wherein the chemical structure of compound **1**, **2**, **3** and **5** are more similar to that of GA-A and GA-D, because the branch chain of substituent of C-17 is a straight chain, including carboxyl, hydroxyl, or methyl ester, which might be the common structural basis of CDK2 activities, while, the branch chain of compound **4** had two oxygenated quaternary carbons (C-23 and C-17) instead of the carbonyl (C-23) and methyl (C-17) in compound **3**, and the branch chain of compound **6** had also no carbonyl or hydroxyl (C-23), which may be the cause of poor biological action to CDK2. 

**Figure 2:**
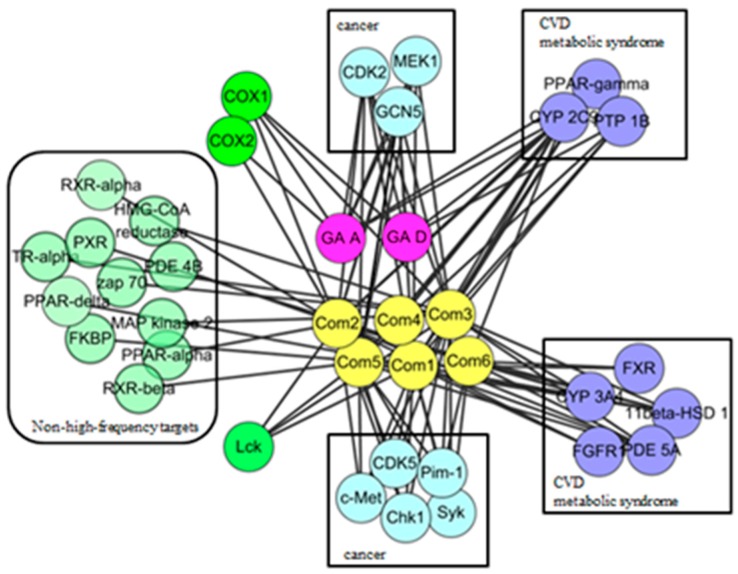
Ingredient-target network of GTs.

In order to observe the biological effect of GTs visually, ingredient-target network of GTs was constructed in Figure 2. According to the classes of corresponding diseases and the number of interactive compounds for each target, 19 high frequency targets were obtained, which could interact with more than one compound. As shown in the top of network, eight targets interacted with GA-A and GA-D could also be hit by the other six GTs, including CYP2C9, CDK2, PPAR γ, GCN5, MEK1, PTP1B, COX1, and COX2. It indicated that GTs might have common pharmacological activities related to cancer, cardiovascular diseases and metabolic syndrome. The 11 specific high-frequency targets of six isolated GTs were shown in the bottom of network, which included the cancer-related targets (CDK5, c-Met, Pim-1, Chk1, and Syk), metabolism-related targets (FXR, CYP3A4, 11beta-HSD1, FGFR1, PDE5A) and immune-related targets of Lck. These 11 specific targets of six isolated GTs might suggest specific pharmacological activities of GTs based on this extraction process. Other non-high-frequency targets might indicate miscellaneous pharmacological activities of GTs, which needed to analyze each ingredient-target interaction of a single GT. A brief description of high frequency targets was given in Table 2.

### 2.3. Protein Interaction Network Analysis of GTs

**Figure 3:**
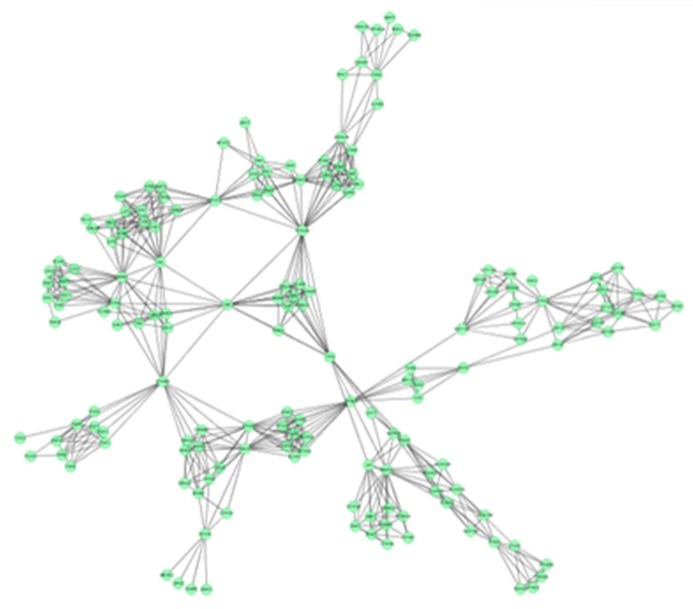
PIN of GTsbased on 19 frequency targets with 185 nodes (proteins) and 733 edges (relations).

**Figure 4:**
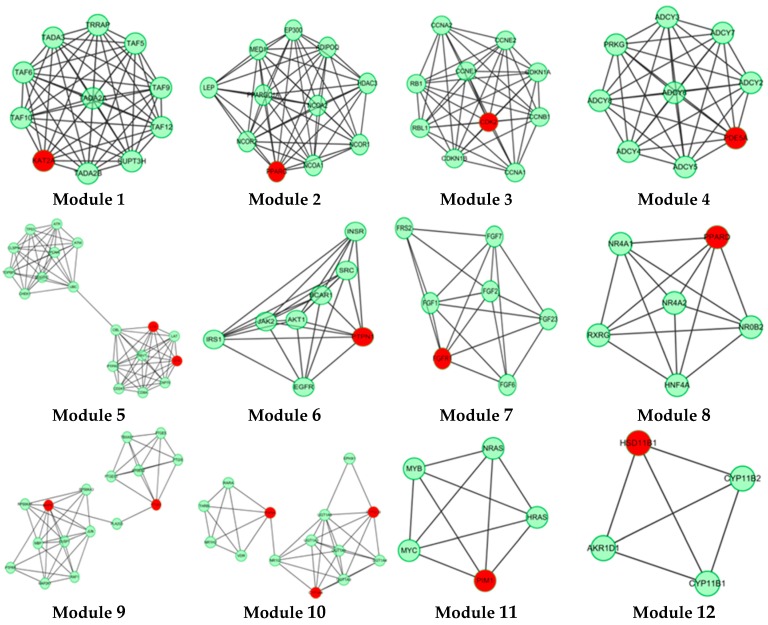
A set of 12 modules were identified from the PIN of GTs.

In this study, 19 high frequency targets were used to construct PIN (Figure 3) for further predicting the anti-cancer activity of GTs. The generated network contained 185 nodes (proteins) and 733 edges (relations). In order to cluster the pharmacological activity of GTs, 12 modules (Figure 4) were identified from PIN by MCODE algorithm. Functional enrichment analysis by BinGO method was shown in Table 3 to annotate potential biological function of the proteins in each module. The results showed that GTs played an essential role in pharmacodynamics with the biological processes, such as regulation of the cell division cycle, lipid metabolic process, protein acetylation, apoptotic signaling pathway, and so on. Half of the modules were related to cancer, including module 1, 3, 6, 7, and 11.

Histone acetylation (HAT) of module 1 was closely related to cancer, including KAT2A, TAF10, TAF9, TADA2A, TADA3, and so on. KAT2A is the gene name of GCN5, which is a typical transcriptional cofactor and requirement for HAT [34]. GCN5 could form multiple complexes by TAFs, including TAF9 [35,36], TADA2A, TADA3 [37], which is quite essential to cancer and HAT. TAFs are important to regulate the cellular differentiation and the speed of cancer cell migration [37]. TAFs are also the important part of transcription factor complex IID (TFIID), which could regulate and control cellular totipotency and differentiation in cancerous and normal cells. Therefore, GTs might regulate HAT by acting GCN5, which was beneficial to inhibit tumor proliferation and metastasis. 

Module 3 showed anti-cancer activity including CCNE1, CCNB1, RBL1, RB1 and CDK2. Cyclin-dependent kinase-2 (CDK2) is an important member in protein kinase family, which could regulate division cycle in eukaryotic cell [38]. Previous study had demonstrated that overexpressing CDK2 would lead to abnormal regulation in cell cycle, which was the important feature for overexpression of cancer cells. CDK2 could also combine with cyclin (gene name: CCN) and phosphorylate downstream targets RB1 and RBL1 [39,40]. Therefore, CDK2 inhibitors are essential for anti-cancer treatment. Based on the pharmacophoric profiling of GTs, GTs are possible to inhibit CDK2 activity for downregulating DNA replication and mitotic cell cycle.

Module 6 and module 7 indicated that GTs might play an important role in anti-tumor effect by regulating receptor tyrosine kinase signaling pathway, especially fibroblast growth factor receptor (FGFR) signaling pathway. Module 11 showed that GTs might inhibit the negative regulation of apoptosis by activating the serine/threonine protein kinase (Pim1), which could promote the apoptosis of tumor cells.

## 3. Materials and Methods 

### 3.1. Plant Material

The fruiting bodies of *G. lucidum* were collected from Hainan in February 2013. The samples were authenticated by Professor Chun-Sheng Liu (School of Chinese Materia Medica, Beijing University of Chinese Medicine, Beijing, China). A voucher specimen (No.130201) has been deposited at the 513 lab of School of Chinese Pharmacy, Beijing University of Chinese medicine.

### 3.2. Extraction

The dried fruiting bodies of *G. lucidum* (30 kg) were milled and soaked into 240 L 70% ethanol in the ratio of 1:8 (*w*/*v*). The mixtures were left for 12 h at room temperature, and then refluxed for three times, each time for two hours. The extract was filtered through a Whatman number 3 filter paper and concentrated with the rotary evaporator (Shanghai Yarong Biochemical Instrument Company, Shanghai, China) at 55 °C to obtain the crude 70% ethanol extract of *G. lucidum*. The crude extract was dissolved in water (12 L), and extracted with ethyl acetate (12 L) and n-butyl alcohol (12 L) for three times, respectively. The extract was filtered and concentrated, yielded an ethyl acetate fraction (463 g, dry weight) and an n-butyl alcohol fraction (160 g, dry weight).

### 3.3. Isolation 

The ethyl acetate fraction was subjected to silica gel (500 g, 200–300 μm, 150 × 10 cm, Qingdao Haiyang Chemical Co., Ltd., Qingdao, China) dry column chromatography using CHCl_3_:EtOAc (4:1) as the eluent, yielded 40 fractions. The 40 fractions were then combined to five groups (F1 to F5) according to the thin layer chromatography (TLC) (20 cm × 20 cm, Qingdao Haiyang Chemical Co., Ltd.).

The F1 fraction (20.0 g) was fractionated into four groups (F_1_1 to F_1_4) with petroleum ether: EtOAc (100:0, 90:10, 80:20, 70:30, 60:40, 50:50, 40:60, 30:70, 20:80, 10:90, 0:100) according to the TLC. F_1_1 and F_1_2 were applied to precipitation crystallization, filtering, recrystallization to obtain compounds **23** and **24**. F_1_3 was subjected to CHCl_3_: EtOAc (2:1) as the eluent, then applied to preparation TLC (Qingdao Haiyang Chemical Co., Ltd.) with petroleum ether: chloroform: methanol (5:5:1) as mobile phase to obtain compound **9**. F_1_3 was subjected to a silica gel column chromatography using CHCl_3_:MeOH (5:1) as mobile phase to obtain compound **10**.

The F2 fraction (78 g) was fractioned into three groups with CHCl_3_: MeOH (80:1, 40:1, 20:1, 9:1, 8:2 , 7:3, 1:1, 3:7 and 0:1) according to the TLC. F_2_1 was rechromatographed over silica gel column using petroleum ether: EtOAc (1:1) as mobile phase to obtain compound **6**. F_2_2 was also subjected to a silica gel column using CH_2_Cl_2_: MeOH (1:1) as the eluent, and further applied to Sephadex LH-20 column (Merck, Darmstadt, Germany), ODS-A (20–45 μm; Merck, Darmstadt, Germany) and MCI-gel CHP-20-P (75–150 μm; Mitsubishi Chemical Co., Tokyo, Japan) with a step gradient of MeOH/H_2_O to yield compounds **11**, **12**, **13**. 

Fraction F3 (55.0 g) was subjected to a silica gel column (75 g, 200–300 μm, 33 cm × 8 cm, Qingdao Haiyang Chemical Co., Ltd.) and eluted with a step gradient of CH_2_Cl_2_/MeOH (80:1, 40: 1, 20:1, 10:1, 9:1, 7:1, 4:1, 1:1, 0:1) to yield seven groups (F_3_1–F_3_7). F_3_1was submitted to column chromatography using CH_2_Cl_2_:MeOH (3:1) as mobile phase to yield compound **4**. F_3_3 and F_3_4 were further subjected with Sephadex LH-20, ODS-A and MCI-gel CHP-20-P, and eluted with a step gradient of MeOH:H_2_O to obtain compounds **1**, **2**, **5**, **8**. F_3_3, F_3_4, and F_3_5 were subjected to a silica gel chromatography eluted with a CH_2_Cl_2_:MeOH:H_2_O (30:1:0–8:2:0.2), and further reapplied to Sephadex LH-20, ODS-A, and MCI-gel CHP-20-P column, and using MeOH as eluting solvents to yield compounds **14**, **16**, **17**, **18**, **19**, **15**. The F_3_4 fraction (10 g) was subjected to Sephadex LH-20 and MCI-gel CHP-20-P open column using MeOH as eluting solvents to yield compound **3**.

**Figure 5:**
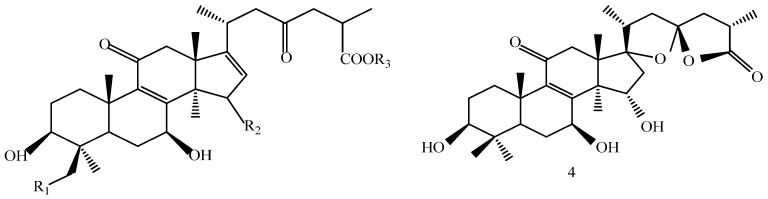

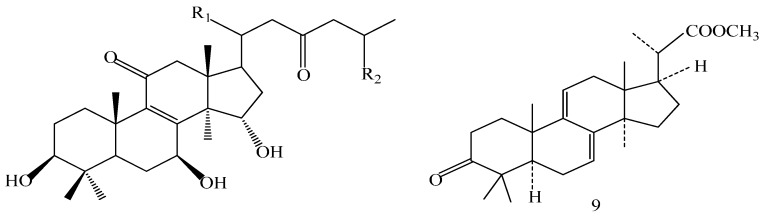

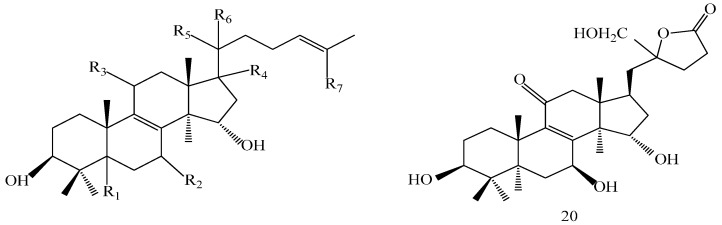

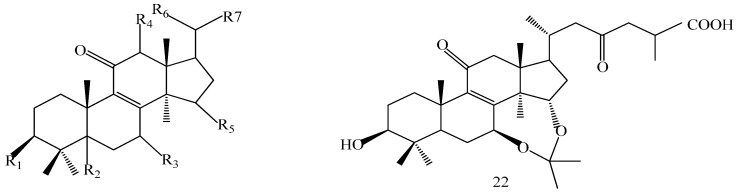

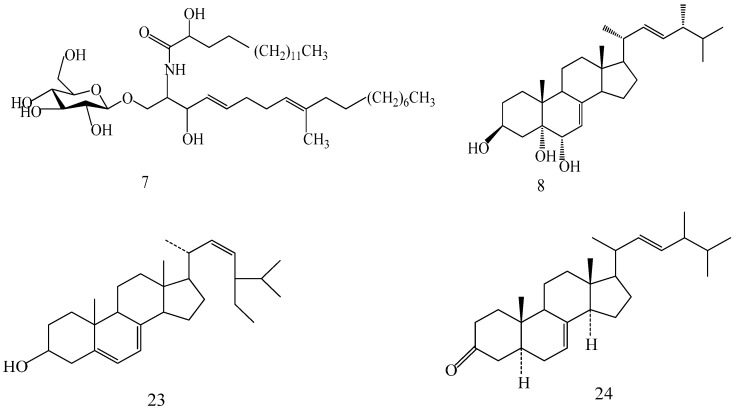
Structures of compounds **1**–**24**.

A 160 g sample of n-butyl alcohol extract was fractionated into three groups according to the TLC with EtOAc: MeOH in the following ratios of 20:1, 10:1, 7:1, 5:1, 3:1, 1:1. Fraction 1 and 3 were applied to silica gel column using a step gradient of CH_2_Cl_2_/MeOH/H_2_O (40:1:0–7:3:0.5) as mobile phase, and further submitted to Sephadex LH-20, ODS-A, and MCI-gel CHP-20-P, and eluted with MeOH/H_2_O to yield compounds **7**, **20**, **21**, **22**. The structures of compounds **1**–**24** are shown in Figure 5.

### 3.4. Reverse Target Identification

Ligand profile module was utilized to reversely identify targets for six isolated and two known positive GTs in Discovery Studio 4.0 (DS, Accelrys Inc., San Diego, CA, USA). Previous researches indicated GA-A and GA-D have the clear anti-cancer activity [18,41]. Therefore, in this paper, GA-A and GA-D were selected to be the typical positive compounds for guiding the anti-cancer bioactive prediction of six isolated GTs. Eight compounds were first minimized in CHARMm force field with MMFF 94 partial charge. Diverse conformations of 8 GTs were constructed by the BEST method within 255 conformations, and the relative energy threshold was set to 20.0 kcal/mol.

Eight GTs were *in silico* profiled by structure-based pharmacophore (SBP) models database called pharmaDB in DS [42,43,44]. In order to evaluate the predict activity of GTs, two indexes were chosen to judge the predicted results. The initial judgment criterion was the overlap degree of molecules and pharmacophore, which was represented by the fit value [45]. Fit value greater than 0.5 indicated better-quality conformational coverage between compounds and SBP, which suggested potential biological activity of compounds for corresponding target. The second judgment criterion was the hit number of SBP models, which could to some extent reveal the biological activity of compounds and importance of the target. As SBP models were constructed by the crystal structure in the RCSB protein data bank (PDB), one target might have more than one crystal structure, and meanwhile one crystal structure could produce several SBP models. Therefore, a single target could have multiple SBP models, which represented various binding modes between ligands and receptors [46]. According to the two indexes, the results of predicted targets and interaction were obtained with the fit value more than 0.5 and the rank of hit number of SBP models. Based on the result of reverse target identification, ingredient-target network of GTs was structured in Cytoscape 3.2.0 (Cytoscape Consortium, San Diego, CA, USA) [47]. The high-frequency targets and pharmacological action of GTs were predicted and validated by the analysis of network nodes and edges.

### 3.5. Network Construction and Analysis

The PPI information of high-frequency targets was derived from the online database of String 9.1 [48] which could provide experimental and predicted PPI information. Confidence scores could be provided from String, which represented the stable degree of interaction. In this paper, the PPIs of high-frequency targets, with a confidence score higher than 0.9, were collected to construct PIN by Cytoscape 3.2.0. Then, union calculation was implemented and duplicated edges of PPIs were removed by Advanced Network Merge [49]. Finally, the largest connected sub-graph was obtained as the PIN of GTs.

A functional module is the structural unit of PIN, which can represent cellular functional organization and extract meaningful information from the complex PIN. In this paper, functional modules with more than four sides were identified by the MCODE methods [50]. Based on the identified modules of GTs, GO enrichment analysis was used to predict possible biological processes of the identified modules using the BinGO method [51]. Wherein, *p*-value was utilized to represent the probability that a group of genes was from the same gene ontology, and less *p*-value indicated more probability.

## 4. Conclusions

*G. lucidum* is one of the most commonly used TCM for thousands of years. The discovery of therapeutic basis and action mechanism of *G. lucidum* was essential to clinical use of drugs from *G. lucidum*. In this paper, an innovated research mode of extraction, isolation, pharmacological prediction, and PIN analysis was presented to discover the novel compounds and predict the anti-cancer activity of *G. lucidum*. Twenty-four compounds were isolated and identified from the 70% ethanol extract of the fruiting bodies of *G. lucidum*, including nineteen triterpenes, three steroids, one cerebroside, and one thymidine. Six GTs (Compounds **1**–**6**) were first reported from the species of *G. lucidum*. One cerebroside and one steroid were first isolated from the genus *Ganodema*. Then, pharmacophoric profiling of these six GTs was implemented to analyze their potential therapeutic targets. Nineteen high-frequency targets were obtained and mainly related to cancer and metabolic syndrome, especially CDK2 and PPAR γ. PIN and modules analysis of the 19 high frequency targets indicated that the cell division cycle regulating and protein acetylation might be the main anti-cancer mechanism of these six GTs. In this paper, molecular simulation and PIN analysis were directly utilized to research the potential activity of isolated compounds from the source of extraction of TCM. Actually, the results also need to be validated by wet experiments. However, compared to biochemistry and animals experiment, it might be a rapid, economical, and efficient method for discovering potential pharmacological activity of new isolated compounds.

## Figures and Tables

**Table:** 

Compound	R_1_	R_2_	R_3_
**1**	H	β-OH	CH_3_
**2**	H	β-OH	H
**3**	-OH	α-OH	H

**Table:** 

Comp.	R_1_	R_2_
**5**	α-CH_3_	OH
**16**	α-CH_3_	COOH
**17**	β-CH_3_	COOCH_3_
**19**	α-CH_3_	COOCH_3_
**21**	β-CH_3_	COOH

**Table:** 

Comp.	R_1_	R_2_	R_3_	R_4_	R_5_	R_6_	R_7_
**6**	β-H	α-OH	=O	β-H	α-CH_3_	β-H	CH_3_
**13**	α-H	=O	H	α-H	α-CH_3_	β-H	COOH
**15**	α-H	β-OH	=O	α-H	β-CH_3_	β-OH	COOH
**18**	β-H	β-OH	=O	β-H	α-CH_3_	β-H	COOH

**Table:** 

Comp.	R_1_	R_2_	R_3_	R_4_	R_5_	R_6_	R_7_
**10**	β-OH	α-H	β-OH	β-OH	=O	β-CH_3_	(CH_2_)_2_COOH
**11**	=O	α-H	β-OH	β-H	α-OH	α-CH_3_	=O
**12**	β-OH	α-H	=O	β-H	=O	α-CH_3_	=O
**14**	β-OH	β-H	β-OH	β-H	α-OH	α-CH_3_	CH_2_OH

**Table 1:** Top 20 interaction information of GTs and targets.

Compound	Target	Fitvalue	Hit *	Compound	Target	Fitvalue	Hit *
Com3	CDK2	0.891	13	Com6	CYP3A4	0.792	5
Com2	CDK2	0.817	12	Com5	CYP3A4	0.763	5
Com5	CDK2	0.824	11	GA-A	CDK2	0.753	5
Com1	CDK2	0.796	11	Com3	11beta-HSD 1	0.83	4
Com2	PPAR γ	0.787	10	Com2	COX1	0.824	4
Com3	PPAR γ	0.789	7	Com3	PTP 1B	0.724	4
Com5	PPAR γ	0.833	6	Com2	c-Met	0.722	4
Com2	CYP3A4	0.909	5	Com2	Lck	0.666	4
Com1	CYP3A4	0.902	5	Com3	PPAR *γ*	0.635	4
Com3	CYP3A4	0.883	5	GA-D	CDK2	0.707	3

* Hit is the hit number of SBP models from one target.

**Table 2:** Predicted target information of GTs.

ID	Target	Number of Compounds	Gene Name	Possibly Relevant Diseases
1	CYP2C9	**8**	CYP2C9	ADMET
2	CDK2	**6**	CDK2	cancer
3	GCN5	**6**	KAT2A	cancer
4	PPAR γ	**5**	PPARG	obesity
5	COX1	**5**	PTGS1	pain, cardiovascular diseases
6	MEK1	**5**	MAP2K1	cancer
7	CYP3A4	**5**	CYP A4	ADMET
8	11beta-HSD 1	**5**	HSD11B1	diabetes, obesity
9	PDE5A	**5**	PDE 5A	cardiovascular diseases
10	PTP1B	**4**	PTPN1	diabetes, obesity
11	Syk	**4**	Syk	cancer
12	Lck	**4**	Lck	autoimmune suppression
13	c-Met	**4**	MET	cancer
14	Chk1	**4**	Chk1	cancer
15	Pim-1	**4**	Pim-1	cancer
16	CDK5	**3**	CDK5	cognitive defects, cancer
17	COX2	**2**	Ptgs2	pain, inflammation
18	FXR	**2**	NR1H4	ADMET, bile acid-induced hepatotoxicity
19	FGFR1	**2**	FGFR1	diabetic retinopathy, cardiovascular diseases, cancer

**Table 3:** GO biological process terms of the modules display partially.

Modules	*p*^−^Value	GO Terms
Module 1	1.69 × 10^−21^	histone acetylation
Module 2	1.83 × 10^−13^	cellular lipid metabolic process
Module 3	1.89 × 10^−18^	interphase of mitotic cell cycle
Module 4	1.47 × 10^−22^	activation of protein kinase A activity
	7.75 × 10^−20^	cellular response to glucagon stimulus
Module 5	1.88 × 10^−12^	antigen receptor^−^mediated signaling pathway
	1.21 × 10^−11^	positive regulation of response to stimulus
Module 6	7.82 × 10^−14^	transmembrane receptor protein tyrosine kinase signaling pathway
Module 7	1.57 × 10^−17^	fibroblast growth factor receptor signaling pathway
Module 8	1.03 × 10^−13^	transcription initiation from RNA polymerase II promoter
Module 9	8.98 × 10^−17^	prostaglandin biosynthetic process
Module 10	1.11 × 10^−15^	xenobiotic metabolic process
Module 11	1.30 × 10^−07^	positive regulation of Rac protein signal transduction
	4.46 × 10^−06^	negative regulation of apoptotic process
Module 12	1.02 × 10^−10^	glucocorticoid biosynthetic process
